# Supplementary material for: Nurses’ perspectives on their communication with patients in busy oncology wards: A qualitative study
Source: PLoS One. 2019 Oct 24;14(10):e0224178. doi: 10.1371/journal.pone.0224178 (PMC6812861; doi:10.1371/journal.pone.0224178)
Supplement: S1 Table — (DOCX) [file pone.0224178.s001.docx]

**S1 Table. Sample findings from the parent study to stimulate discussion**

|  | **Descriptions** |
| --- | --- |
| **Example 1** | There was an interaction between a nurse and a patient during the routine of administering medication. The nurse gave the patient her analgesic drug and told the patient to call if she felt unwell or needed more pain medication during the night. The nurse then went on to ask the patient if the analgesic drug that the patient had been given before physiotherapy had been effective. The patient responded in the affirmative, then said that her ‘legs are weak … floating’. The nurse then explained to the patient that her being in bedrest for  the past few days might be the issue, and that the patient therefore needed to build up her strength. That was the reason why the nurse gave the patient her analgesic drug each time before physiotherapy so that the patient’s pain could be better controlled for her to re-build her strength. The patient thanked the nurse, indicating that the explanation was helpful, and that she now felt less worried about her legs. |
| **Example 2** | Another nurse had administered the medication to a patient and moved on to other patients, she noticed that the patient took out his own medication from a bag. The nurse immediately stopped the patient, using a very harsh tone. In response, the patient explained that she was not trying to take her own pill in addition to the medication that she had received; rather, she wanted to compare the pill that she had just received with the one that she had previously been prescribed, since she thought that the pill that she had received looked a little different. After the patient’s explanation, the nurse realized that she had mistaken the patient’s intent. She walked away without saying anything, but the patient was not pleased. |
