# Supplementary material for: Nurses’ perspectives on their communication with patients in busy oncology wards: A qualitative study
Source: PLoS One. 2019 Oct 24;14(10):e0224178. doi: 10.1371/journal.pone.0224178 (PMC6812861; doi:10.1371/journal.pone.0224178)
Supplement: S2 Table — (DOCX) [file pone.0224178.s002.docx]

**S2 Table. An anonymized table of nurses’ demographic characteristics and backgrounds in oncology care**

|  | N=11 |
| --- | --- |
| **Sex**  Male  Female | 1 (9.1%)  10 (90.9%) |
| **Age (years)**  29 or below  30-40  41-50 | 7 (63.6%)  2 (18.2%)  2 (18.2%) |
| **Education**  BSc in Nursing only  Master of Nursing only  BSc Nursing and Cert^a^  Master of Nursing and Cert^a^ | 6 (54.5%)  3 (27.3%)  1 (9.1%)  1 (9.1%) |
| **Years of work experience in nursing**  1-2  3-5  >5 | 1 (9.0%)  5 (45.5%)  5 (45.5%) |
| **Years of work experience in oncology**  1-2  3-5  >5 | 1 (9.0%)  5 (45.5%)  5 (45.5%) |

a Certificate in palliative or oncology nursing
