# Supplementary material for: Nurses’ perspectives on their communication with patients in busy oncology wards: A qualitative study
Source: PLoS One. 2019 Oct 24;14(10):e0224178. doi: 10.1371/journal.pone.0224178 (PMC6812861; doi:10.1371/journal.pone.0224178)
Supplement: S1 File — (DOCX) [file pone.0224178.s003.docx]

**S1 File. Interview guide**

1. What are the pleasures/challenges/difficulties that you have encountered when working in oncology?
2. How does the work environment influence your work in the oncology ward?
3. What are the needs and concerns of cancer patients, and how do you respond to them?
4. How do you prioritize the concerns of patients? In your perspective, what are the most urgent and less urgent needs of patients?
5. What would you do if you were unable to immediately address the less urgent needs of patients?
6. How do you communicate with patients during procedural care such as admitting patients, administering medications, dressing wounds, and discharging patients? What do you usually talk to patients about during these procedures?
7. When is the opportune time for you to communicate with patients?
8. What constitutes effective communication training for cancer care nurses?
9. Is there anything else that you would like to share?
