# Supplementary material for: Nurses’ perspectives on their communication with patients in busy oncology wards: A qualitative study
Source: PLoS One. 2019 Oct 24;14(10):e0224178. doi: 10.1371/journal.pone.0224178 (PMC6812861; doi:10.1371/journal.pone.0224178)
Supplement: S2 File — (DOCX) [file pone.0224178.s004.docx]

**S1文件：面談指引**

1.在腫瘤科工作有什麼樂趣/挑戰/困難？

2.工作環境如何影響你在腫瘤科病房的服務？

3.癌症患者有什麼需要和關注，你如何回應？

4.你怎樣把患者關心的事情排列優次？你認為什麼是患者最急切和不太急切的需要？

5. 患者有不太急切的需要，但你無法立即處理，你會怎麼做？

6. 在護理患者的過程中（如：接收患者，分派藥物，敷裹傷口和辦理出院），你怎樣與患者溝通及通常與患者談什麼？

7. 何時與患者溝通是適當的時機？

8. 哪些要素有助培訓腫瘤科護士有效地溝通？

9. 你還有什麼想分享？
