# Supplementary figures and images for: Nurses’ perspectives on their communication with patients in busy oncology wards: A qualitative study
Source: PLoS One. 2019 Oct 24;14(10):e0224178. doi: 10.1371/journal.pone.0224178 (PMC6812861; doi:10.1371/journal.pone.0224178)

**S1 Figure. Initial thematic map of the qualitative findings**


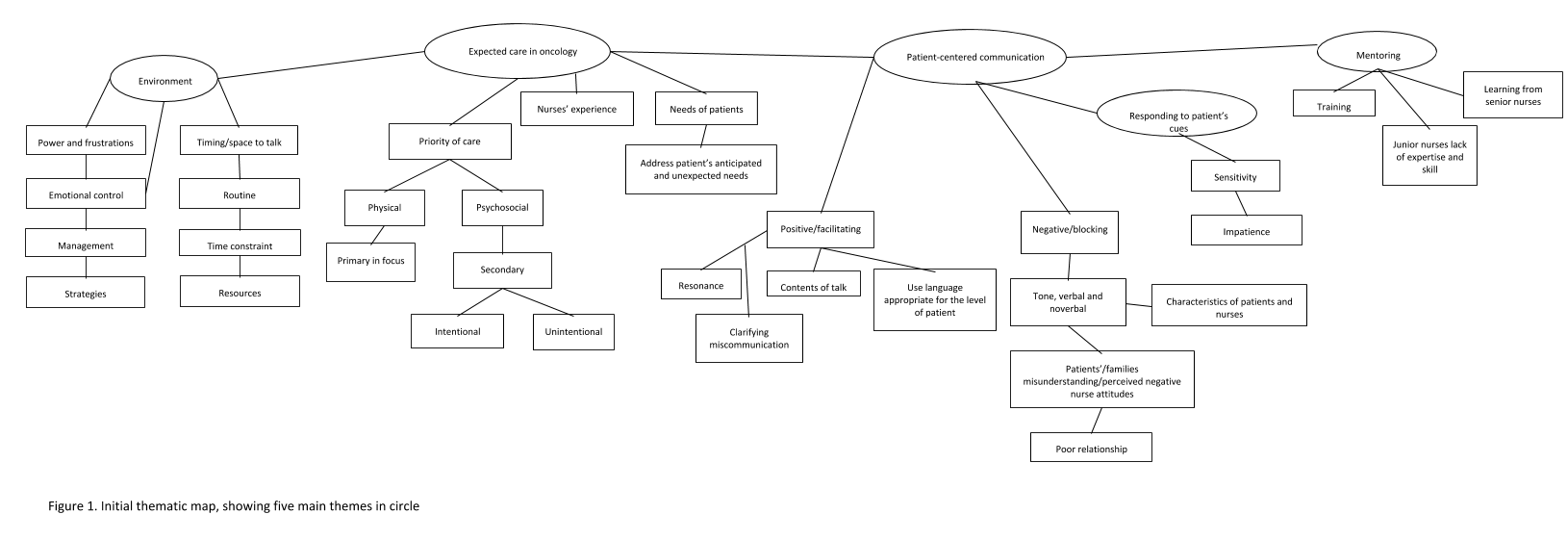

Supplement: S1 Fig — (DOCX) [file pone.0224178.s005.docx]

**S2 Figure. Developed thematic map of the qualitative findings**


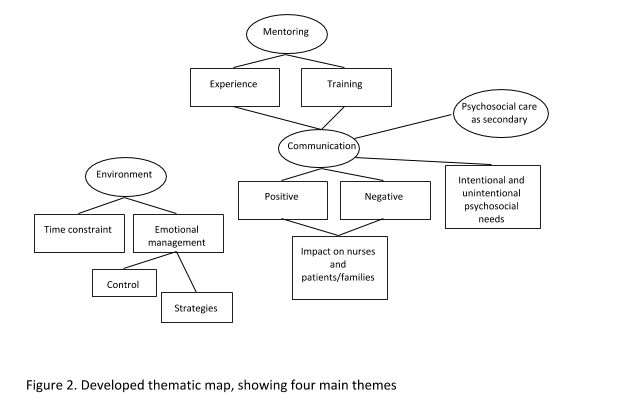

Supplement: S2 Fig — (DOCX) [file pone.0224178.s006.docx]

**S3 Figure. Final thematic map of the qualitative findings**


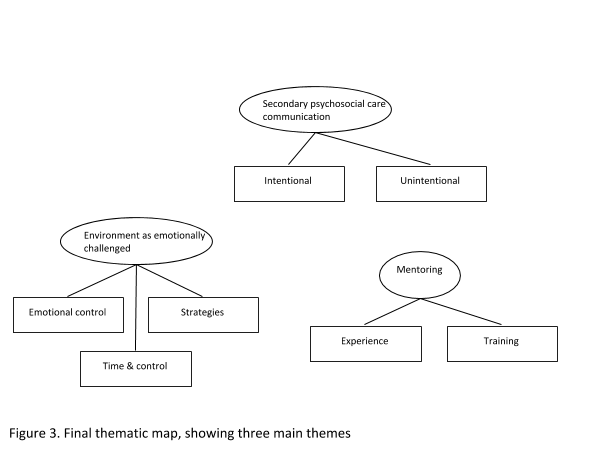

Supplement: S3 Fig — (DOCX) [file pone.0224178.s007.docx]
