# Supplementary material for: Nurses’ perspectives on their communication with patients in busy oncology wards: A qualitative study
Source: PLoS One. 2019 Oct 24;14(10):e0224178. doi: 10.1371/journal.pone.0224178 (PMC6812861; doi:10.1371/journal.pone.0224178)
Supplement: Attachment — Submitted filename: GRF paper responses to reviewers comments PLOS One.docx [file pone.0224178.s008.docx]

**Responses to reviewers’ commments**

| **Reviewers’ comments** | **Responses** |
| --- | --- |
| you may wish to refer to the COREQ guidelines | Thanks for the suggestion, COREQ was used as a guide. |
| Include a copy of the interview guide used in the study, in both the original language and English, as Supporting Information, or include a citation if it has been published previously. | Copies of the interview guide are included as supporting document. |
| Title is reviewed so the focus is nurse-patient communication in oncology wards. There needs to be a clearer focus on skilled communication throughout the essay | Thanks for the suggestion. The title has been revised with a focus on nurse-patient communication in oncology wards. |
| An anonymised table of who the participants are and how long they have worked in oncology should be included. | An anonymized table is included. |
| Reason for why focus groups and interviews are both used in the study as nurses can give different accounts according to which method is used. | Similar to other researchers, both methods were combined for practical considerations, that is individual interviews were offered to accommodate the busy schedule of the nurse participants, who were unable to attend the focus group ([Rees *et.al.*2003](https://onlinelibrary-wiley-com.ezproxy.lb.polyu.edu.hk/doi/full/10.1111/j.1365-2648.2007.04559.x#b41),[Taylor 2005](https://onlinelibrary-wiley-com.ezproxy.lb.polyu.edu.hk/doi/full/10.1111/j.1365-2648.2007.04559.x#b48)). |
| Focus of the article should be on the nurses response to patients' emotions and their own emotional response, which fit with theoretical work related to 'emotional labour' | Thanks for the suggestion, literature on emotional labor and work from Prof. Pam Smith has been reviewed and intergrated into the article. |
| More information is needed on what findings from the first part of the study are relevant to the current study. | More information has been included in the paper. This primarily refers to the use of findings from patients’ perceptions of their nurse-patient communication in the same oncology wards (first part of the study) to stimulate discussion with nurses. After the guide questions, nurses were asked to reflect on the patients’ vignettes and to share their views on nurse-patient communication in the oncology wards. |
| Please see the work of Wilkinson et al related to barriers to communication. | Thanks for the suggestion. Further reading on Wilkinson’s work has been integrated into the discussion. |
| **Reviewers’ comments** | **Responses** |
| More reflexivity is needed on the data collected from the focus groups and the data from individual interviews. | Thanks for the suggestion, reflexivity on the data collected is included. |
| How the data were coded and how psychosocial care, values and emotions were coded from the data. There needs to be explanations of how comparisons were made between the different types of data. More information is needed on what codes were considered significant and patterned these need to be identified. | Thanks for the suggestion. The data process is made more explicit and comparison of different data was provided**.** Further information is provided on the significant codes and identified patterns. |
| A thematic map is referred to again this map needs to be discussed and the process of developing this map further explored. More information is needed on how the final three themes were agreed. | 1. The development of the thematic map is included as figures. Information on how the final three themes were agreed upon is included in the data analysis section. |
| The methodology description does not provide the theoretical framework underpinning the qualitative methodology employed in the study. | Thanks for the comment. A theoretical understanding underpins the use of interviews as a qualitative methodology is included. |
| The description of the conduct of the focus groups is unnecessary and the manuscript would benefit from a description of the questions asked | Thanks for the suggestion. The description of the focus group is removed. |
| The supplementary table only includes a copy of the two scenarios used to orientate participants not the interview schedule. | The interview guide is included. |
| Under participants the authors stated that 11 participants is sufficient to reach theoretical saturation - how was this established? | Thanks for the questions. Changes were made for more clarity and check was made for the saturation. |
| Unclear whether the two reviewers coding the data independently and how differences were reconciled. Overall there needs to be much greater detail provided for the methodology and rigor checks. | Thanks for the comments. More clarity was made to show that two reviewers have coded the data independently and for any discrepancies, they would be discussed at the regular meetings. COREQ is used as the guide to check the process. |
| Results - lines 187-190 - this paragraph does not seem to fit the context of the surrounding text. The presentation of themes are descriptive rather than a thematic analysis and there are too few quotes or discussion of the complexity of different nursing experiences. | The paragraph has been removed and more quotes were included for the discussion of the complexity of different nursing experience. |

Rees CE, Ford JE, Sheard CE. Patient information leaflets for prostate cancer: which leaflets should healthcare professionals recommend? Patient Education and Counseling. 2003;49:263-72.

Taylor B. The experiences of overseas nurses working in the NHS: Results of a qualitative study. Diversity in Health and Social Care. 2005;2:17-27.
